# Sub-optimal menstrual materials and vaginal microbiome disruption in women relying on sex for livelihood

**DOI:** 10.3389/fcimb.2025.1662237

**Published:** 2026-01-12

**Authors:** Supriya D. Mehta, Garazi Zulaika, Edyth Osire, Walter Agingu, Souvik Paul, Cynthia Akinyi, Stefan J. Green, Anna M. van Eijk, Runa K. Bhaumik, Fredrick O. Otieno, Penelope A. Phillips-Howard

**Affiliations:** 1 Department of Internal Medicine, Division of Infectious Diseases, Rush University, Chicago, IL, United States; 2 Department of Clinical Sciences, Liverpool School of Tropical Medicine, Liverpool, United Kingdom; 3 Nyanza Reproductive Health Society, Kisumu, Nyanza, Kenya; 4 Division of Epidemiology & Biostatistics, University of Illinois Chicago School of Public Health, Chicago, IL, United States; 5 Genomics and Microbiome Core Facility, Rush University, Chicago, IL, United States

**Keywords:** menstrual health, menstrual hygiene management, menstruation, sex work, vaginal microbiome, vaginal microbiota, bacterial vaginosis, sexually transmitted infection

## Abstract

**Background:**

Sub-optimal menstrual materials (MM), such as using cloths, cotton balls, or tissues, can adversely affect the vaginal microbiome (VMB). Women who rely on sex for economic livelihood often use sub-optimal materials to conceal menstruation and avoid loss of income. We hypothesized that among women who rely on sex for economic livelihood, those using sub-optimal MM would be more likely to have non-optimal VMB than those with adequate MM.

**Methods:**

This cross-sectional analysis used baseline data from women participating in a trial assessing the impact of reusable menstrual discs on the VMB, Bacterial vaginosis (BV), and sexually transmitted infections (STIs). Data on sociodemographics, menstrual materials, and sexual practices were collected via interviewer-administered survey. Clinician-collected vaginal samples were tested for BV, STI, and VMB. VMB was assessed via 16S rRNA gene amplicon sequencing. A suite of statistical approaches identified factors associated with sub-optimal MM (use of cotton balls, tissue, or cloth) and VMB composition.

**Results:**

407 women were enrolled February through October 2023, with median age 27 years, 24.7% were HIV-positive, 42.2% had BV, and 21.9% had STI (composite of chlamydia, gonorrhea, trichomoniasis). Vaginal community state type (CST) was primarily diverse (CST-IV; 63.5%), or *Lactobacillus iners* dominated (CST-III; 28.1%), while CST-I (*L. crispatus* dominated) was uncommon (7.9%). Sub-optimal MM was reported by 42.0% of participants and in multivariable modeling, was more common among women with indicators of economic strain. In multivariable analyses, alpha diversity was higher with sub-optimal MM and indicators of economic strain. Sub-optimal MM was associated with CST-IV in crude analyses but was attenuated and non-significant when adjusted for age, educational attainment, amount paid at last sexual encounter, number of sex partners, and HSV-2. Non-targeted machine learning algorithms identified non-optimal VMB taxa with greater relative abundance among women with sub-optimal MM.

**Discussion:**

Sub-optimal menstrual materials were used commonly and associated with non-optimal VMB composition. Reusable menstrual discs that may be worn during sex may address the economic factors driving sub-optimal MM that are associated with non-optimal VMB.

## 1 Introduction

HIV and sexually transmitted infections (STIs) remain a global pandemic, with an estimated 40 million persons living with HIV and an estimated 1.65 million new cases of HIV and 374 million cases of curable STIs occurring each year worldwide (Carter et al., 2024; Gottlieb et al., 2024). These infections disproportionately affect economically vulnerable women (Krishnan et al., 2008; Sia et al., 2020), and especially those who rely on sex for livelihood. In a global meta-analysis, the pooled HIV prevalence among women engaged in transactional sex for livelihood (i.e., female sex workers (FSW)) was 30.7%, equating to 11.6-fold higher odds of HIV as compared to general population women (Baral et al., 2012).

Women who rely on sex for livelihood often continue to have sex during menses to meet financial needs. For example, among 1,640 FSW in Nairobi, Kenya, sex during menses was common, reported by 40% (McKinnon et al., 2015). Sub-optimal materials to manage menses – such as using cloths, reusable pads that are insufficiently washed and dried, wearing pads too long, or not having access to soap – are associated with increased risk of reproductive tract infections (Balamurugan and Bendigeri, 2012; Anand et al., 2015; Das et al., 2015; Torondel et al., 2018; Ademas et al., 2020; Al Karmi et al., 2024; Zahra et al., 2025), though studies have not examined the not fit-for-purpose intravaginal methods reported by FSWs in relation to biologically measured outcomes. In our study of 436 secondary schoolgirls in western Kenya randomized to receive either menstrual cups or standard practice (typically reusable pads), those who were using cloth to manage menses at baseline were more likely to have a non-optimal vaginal microbiome (VMB) (Mehta et al., 2021; Mehta et al., 2021), and over 30 months follow-up, girls randomized to receive menstrual cups had 24% reduced odds of BV and 37% increased odds of having a *Lactobacillus*-dominant VMB (Mehta SD et al., 2023).

To address the intersecting challenges of managing menses for women who rely on sex for livelihood, we are undertaking a single arm trial to assess the preliminary efficacy signal of the impact of menstrual discs that can be worn during sex on the VMB, Bacterial vaginosis (BV), STIs, and safety (Clinicaltrials.gov NCT05666778) (Zulaika et al., 2024). Here, we present baseline results in which we: (1) describe sexual practices during menstruation, menstrual hygiene management (MHM), and factors associated with sub-optimal menstrual materials (MM); and (2) examine the association between sub-optimal MM and VMB composition. We hypothesized that participants with sub-optimal MM would be more likely to have non-optimal VMB composition.

## 2 Methods

This study was approved by the institutional review boards of Jaramogi Odinga Oginga Teaching and Referral Hospital (JOOTRH 657-22), and Rush University Medical College (IRB1-22040505), and received favorable opinion from Liverpool School of Tropical Medicine (LSTM, 22-076), and non-human subjects determination from University of Illinois Chicago for having no human subjects contact and only receiving de-identified data (UIC, 2023-0053).

### 2.1 Study design, sample size, and participants

This study used baseline data and biological specimens from the POWWeR Health Study (Periods: Optimizing Working Women’s Reproductive Health), an open-label, single arm trial assessing the impact of soft, disc-shaped menstrual cups on BV and adverse events (ClinicalTrials.gov NCT03051789) (Zulaika et al., 2024). The VMB, BV, and STIs, are measured among participants under usual practice conditions for one year, followed by provision of reusable soft disc-shaped menstrual cups that can be worn during sex, and then followed for VMB, BV, and STIs for another one year. A sample size of 402 participants was sought to be able to detect a 20% decrease in occurrence of BV between the control and intervention phases, and a 1% serious adverse event rate with 80% power (Zulaika et al., 2024).

To be eligible, participants had to be aged 15 to 35 years, having menstruated in the past two months, and having exchanged sex for money or basic necessities (rent/housing, food, medical care for self or children, tuition for self or children) within the past two months, and living and/or working in the Kisumu area. We excluded women who were currently pregnant (as determined by urine hCG test) or had been pregnant in the past six months (as they may still be lactating), or having an intrauterine device (IUD) *in situ* due to risk of expulsion with menstrual cup use. All participants provided written informed consent in their preferred language (English, DhoLuo, Kiswahili).

### 2.2 Data collection

Participants underwent a tablet-based survey administered by female study staff in their language of choice to obtain information on socio-demographics and sexual and menstrual practices. Socio-demographic data included age and socioeconomic and financial indicators. Sexual practices were assessed with numerous variables relating to number of sexual partners, condom use, recency of sex, and payment for sex. Characteristics of the menstrual cycle were assessed as days since last menstrual period, duration of last menstrual period, and menstrual flow (asked as normal, light, or heavy). Phase of menstrual cycle was estimated by adding the days since the last period to the duration in days of the last menstrual period, and then assigned to estimated menstrual phase (Magoutas et al., 2025): 0–5 menstrual, 6–11 follicular, 12–16 ovulatory, 17–42 luteal. Those with missing days since last period or more than 42 days since in the estimated cycle were classified as uncertain. MHM was assessed with several questions including materials used to manage menses, intravaginal practices, and access to soap, water, and privacy at home and during sex work.

Use of sub-optimal menstrual materials was defined as a composite of any use of cloth, cotton balls, or tissue during the last menstrual period. Unsafe intravaginal practices examined: using cloth, tissue, paper, or cotton to wipe inside the vagina to remove fluids between clients, putting something inside the vagina before sexual intercourse to achieve a dry or tight sensation, putting something inside the vagina to keep it dry during menses, use of commercial douche product, and the frequency of wiping inside the vagina during menses, not during menses, and the difference between them (increased, decreased, or the same). We also examined MHM accounting for difficulty accessing water at home or at work, and difficulty accessing privacy at home or at work, in addition to using sub-optimal materials to manage menses.

### 2.3 Specimen collection

All participants underwent a detailed medical history and physical examination by a study clinician. The clinician obtained four vaginal swabs. The first swab obtained was for 16S rRNA gene amplicon sequencing (microbiome), the second for BV, the third for detection of *C. trachomatis* (CT) and *N. gonorrhoeae* (NG), and the fourth for detection of *T. vaginalis* (TV). Vaginal swabs for amplicon sequencing were collected using OMNIgene Vaginal kits (OMR-130; DNA Genotek™), stored at -80 °C until shipment to Chicago for processing. Swabs for amplicon sequencing, CT/NG, and TV were taken immediately to the onsite UNIM Research and Training Laboratory for processing or storage.

### 2.4 Detection of bacterial vaginosis, sexually transmitted infections, HSV-2, and HIV

Following manufacturer protocol, vaginal swabs were tested for CT/NG using the GeneXpert (Cepheid, Sunnydale, California, US). Swabs for TV were processed immediately upon receipt using the OSOM TV antigen detection assay (Sekisui, Lexington, MA, US). Air-dried smears were Gram stained and evaluated for BV according to Nugent’s criteria within 48 hours of receipt (Nugent RP and Hillier, 1991). HIV was assessed on a finger-stick blood sample using Determine rapid assay, following Kenyan national guidelines (Program NASC, 2022). Venous serum specimens were tested for HSV-2 antibody (Kalon HSV-2 immunoglobulin G enzyme-linked immunosorbent assay; Kalon Biological Limited Kingdom) using the manufacturer’s recommended cutoff.

### 2.5 DNA extraction and sequencing

Genomic DNA was extracted using a Chemagic 360 device (Revvity, Hamburg, Germany) and processed for sequencing using a two-stage PCR protocol (Naqib et al., 2018). Full-length 16S rRNA gene sequences were PCR amplified using primers 27F and 1492R, processed using PacBio Kinnex chemistry, and sequenced on a PacBio Revio instrument (Verma et al., 2025) (**Supplementary Materials 1**). Bioinformatics analysis was performed using QIIME2 2023.2 (**Supplementary Materials 1**) (Bolyen et al., 2019). Alpha‐diversity metrics (observed features (Faith, 1992), Shannon Index (Shannon, 1948), Simpson’s Index (Simpson, 1949), and Pielou’s Evenness (Pielou, 1966)) and beta diversity metrics were calculated using q2‐diversity. Taxonomy was assigned to ASVs against multiple databases to find the best match and reported accordingly. Contaminants were identified using the *decontam* program via ASVs in the reagent negative blank controls (Davis et al., 2018). Community state types (CSTs) were identified in a reference dataset using nearest centroid classification (*VA*gina*L* community state typ*E N*earest *C*entro*I*d classifier, (VALENCIA) (France et al., 2020). Raw sequence data (FASTQ files) were deposited in the National Center for Biotechnology Information (NCBI) Sequence Read Archive (SRA), under BioProject identifier PRJNA1279642.

Of the 407 participants, 406 had a sample submitted for VMB assessment, with median sequence depth of 31,220 reads (interquartile range 23,362 – 41,185). Among 406 observations, n=11 (2.5%) had fewer than 2,500 sequence reads and were excluded from VMB analyses. Prior to analyses, data were filtered to retain taxa that contributed at least 0.01% of the total sequence reads, resulting in retention of 111 of 821 taxa. These 111 taxa included 16 “uncultured” and “unidentified” taxa with no phylogeny, which were excluded from inferential analysis as being non-informative, along with seven sparse taxa (having fewer than 10 non-zero observations). The resulting dataset comprised N = 88 taxa.

### 2.6 Statistical analysis

The primary exposure of interest was sub-optimal menstrual materials, defined as using cotton balls, cloth, or tissues to manage the last menstrual period occurring within the past 6 weeks. To identify factors associated with sub-optimal MM, we conducted multivariable Poisson regression with robust variance to estimate prevalence ratios. Next, we examined sub-optimal MM in relation to VMB composition in targeted and non-targeted analyses. Our *a priori* hypothesis was that number of sex partners, types of sex work venue, and sociodemographic indicators could be confounders, being associated with both VMB composition (primary outcome) and sub-optimal menstrual materials (primary exposure).

#### 2.6.1 Targeted analyses

Targeted analyses examined: CST, alpha diversity, and relative abundance of *L. crispatus*. Due to sparsity in CST-II (*L. gasseri* dominated, n=1) and CST-V (*L. jensenii* dominated, n=1), these two observations were excluded from CST analysis. We applied multinomial logistic regression with robust variance to estimate the association between variables of interest and CST, with CST-I being the referent. Multivariable linear regression with robust variance was applied to quantify associations between variables of interest and alpha diversity measures (richness, evenness, Shannon index, Simpson’s index). Notably, in 87.1% of observations, the relative abundance of *L. crispatus* was less than 1%, and this analysis was abandoned. Across outcomes, model selection was aided by minimizing Akaike’s Information Criterion (AIC), with review of similarly performing models (**Supplementary Material 2**). Post estimation, we observed variance inflation factors (VIF) ranged 1.05 – 1.20 for factors associated with poor MM; 1.01 – 3.38 for factors associated with CST; and 1.01 – 1.41 across alpha diversity outcomes, demonstrating no concern for multicollinearity.

#### 2.6.2 Non-targeted analyses

**Figure 1:**
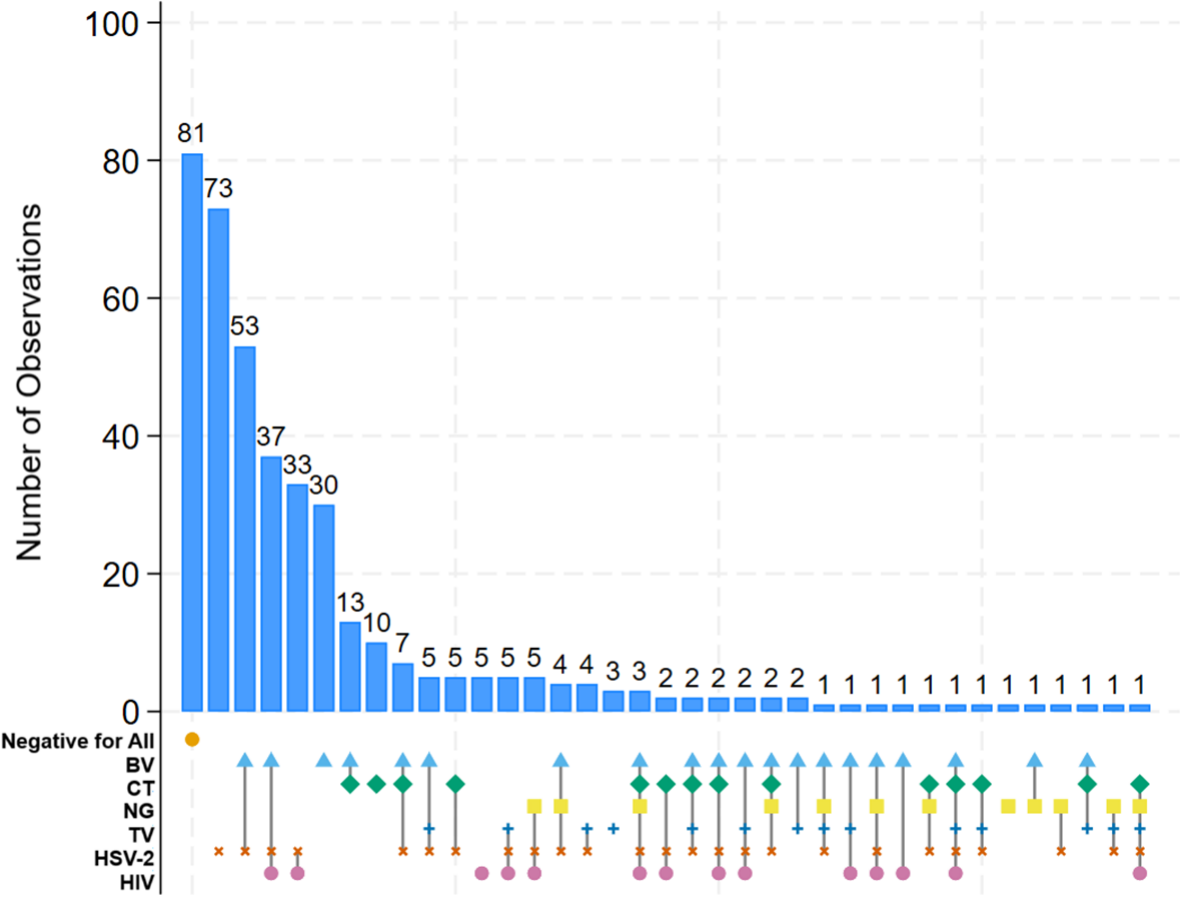
Intersections of bacterial vaginosis, sexually transmitted infections, and HIV. **Figure 1** shows the intersections of BV, STI, and HIV statuses, with the rows of the matrix corresponding to the status, and the columns to the intersections between these statuses. The number of participants observed in the intersection is shown in the bar with the number of participants labelled (y-axis and each bar).

For taxa with at least 1% non-zeros, zeros were imputed using geometric Bayesian multiplicative replacement (*zCompositions* package in R (Palarea-Albaladejo and Martín-Fernández, 2015)). Raw counts were then converted to relative abundances followed by centered log-ratio (CLR) transformation. Among the 88 taxa, we applied Lasso regression using the *glmnet* package in R (version 4.3.1) (Friedman et al., 2021). To identify taxa associated with sub-optimal MM independent of any infection, we estimated models (1) restricted to participants with no infections identified, comparing sub-optimal MM to those with no infections and adequate MM; and (2) among all participants, adjusted for BV, HIV, STI, and HSV-2. Taxa selected by Lasso were used to fit standard logistic regression models, and taxa with p-values less than 0.20 were retained. Following a stability selection approach (Meinshausen and Bühlmann, 2010; Hofner et al., 2015), this procedure was repeated over 100 bootstrap resamples. Stability selection reduces the risk of false positives in high-dimensional data comparisons by keeping only the variables that are consistently selected across many sub-samplings. As recommended by Meinshausen and Bühlmann, we report taxa selected in over 60% of the bootstrap iterations alongside coefficients and p-values, and focus discussion to only the taxa showing the highest percent stability. This was supplemented with comparison of Bray-Curtis similarity to identify taxa contributing most to dissimilarity by sub-optimal MM status, and for each of the following conditions (**Figure 1**): negative for all infections, (1) HSV-2 only, (2) HIV only, (3) BV only, (4) HSV-2 and BV, (5) HSV-2 and HIV, (6) HSV-2 and BV and HIV, (7) BV and STI (composite of NG, CT, or TV due to sparseness of any single etiology), (8) BV and STI and HSV, and (9) other mixed infection status.

## 3 Results

### 3.1 Results of recruitment and eligibility

From February 8^th^ through September 25^th^, 2023, 473 women were screened, and 415 (87.8%) were enrolled. Women could be ineligible for more than one reason; the results of screening and eligibility are detailed in **Supplementary Materials 3**. After informed consenting, there were 8 screening failures: 3 subsequently refused speculum examination, 2 subsequently disclosed being outside the eligible age range, 3 subsequently disclosed being amenorrheic. Thus, the final cohort proceeded with 407 women, with 395 evaluable in relation to VMB.

### 3.2 Characteristics of the cohort

Participants were median age 27 years, and 59.2% with primary education or less (**Table 1**). Half (49.4%) of participants reported past month income of < 10,000 KES (~$77 USD), and 11.6% reported missing a meal in the past 7 days due to not having enough money. In the past 30 days, participants reported a median of 25 (IQR 15-50) clients and median payment at last sex act was 700 KES (median ~$5.50 USD). HIV was common, with 24.7% of women reporting themselves to be infected (no new infections were detected with testing), of whom 84% reported taking ARVs. By laboratory testing, 62.8% were HSV-2 seropositive, 42.2% had BV, and 21.9% had an STI (CT (12.8%), NG (5.4%), and/or TV (7.4%)). The combinations of infections are shown in **Figure 1**. Overall, 20.2% of participants were negative for HIV, BV, and all STIs. Mixed infections were common, most frequently in combinations with HSV-2 seropositivity and BV.

**Table 1:** Distribution of participant characteristics by community state type (1).

Variables	Total N=393 n (%)	CST-I, N = 31 n (%)	CST-III, N = 111 n (%)	CST-IV, N = 251 n (%)	p-value				
Demographics									
Age group									
15–26 years	194 (49.4)	22 (11.3)	61 (31.4)	111 (57.2)	**0.007**
27–35 years	199 (50.6)	9 (4.5)	50 (25.1)	140 (70.4)	
Educational attainment									
Less than secondary school	232 (59.0)	10 (4.3)	65 (28.0)	157 (67.7)	**0.005**
Secondary school or higher	161 (41.0)	21 (13.0)	46 (28.6)	94 (58.4)	
Earns income outside of sex work									
No	226 (57.5)	22 (9.7)	59 (26.1)	145 (64.2)	0.205
Yes	167 (42.5)	9 (5.4)	52 (31.1)	106 (63.5)	
Income earned in past one month									
<5,000 KSH	32 (8.1)	1 (3.2)	6 (18.8)	25 (78.1)	0.570
5000 - <10,000 KSH	164 (41.7)	14 (8.5)	46 (28.1)	104 (63.4)	
>10,000 (2)	197 (50.1)	16 (8.1)	59 (30.0)	122 (61.9)	
Missed a meal in the past 7 days due to lack of money									
No	349 (88.8)	31 (8.9)	104 (29.8)	214 (61.3)	**0.005**
Yes	44 (11.2)	0 (0)	7 (15.9)	37 (84.1)	
Sexual practices									
Currently have a non-paying main partner, boyfriend, husband									
No	116 (29.8)	8 (6.9)	22 (19.0)	86 (74.1)	**0.030**
Yes	276 (70.2)	23 (8.3)	88 (31.9)	165 (59.8)	
Median (IQR) number of sex partners…									
The last day worked	2 (1-3)	2 (1-3)	2 (1-3)	2 (1-3)	0.507
The last 7 days	10 (4-18)	6 (4-15)	10 (4-15)	10 (5-20)	0.133
The last 30 days	25 (15-50)	15 (10-40)	20 (10-40)	30 (15-50)	**0.010**
In general, more clients who are regular, casual, or new									
Regular	53 (13.5)	3 (5.7)	19 (35.8)	31 (58.5)	0.778
Casual	5 (1.3)	0 (0)	1 (20.0)	4 (80.0)	
New	23 (5.9)	1 (4.3)	6 (26.1)	16 (69.6)	
Similar mix of all three	312 (79.4)	27 (8.6)	85 (27.2)	200 (64.1)	
Where meet clients (not mutually exclusive) (3)									
On the street	254 (64.6)	24 (9.5)	75 (29.1)	156 (61.4)	0.213
Meet in a home	28 (7.1)	4 (14.3)	10 (35.7)	14 (50.0)	0.17
Meet at a truck stand	32 (8.1)	3 (9.4)	10 (31.3)	19 (59.4)	0.798
Brothel/sex den	107 (27.7)	4 (3.7)	22 (20.6)	81 (75.7)	**0.008**
Bar/restaurant/club	279 (71.0)	22 (7.9)	80 (28.7)	177 (63.4)	0.941
Lodge, guest house	211 (53.7)	20 (9.5)	63 (29.9)	128 (60.7)	0.271
Meet in social gatherings	32 (8.1)	5 (15.6)	9 (28.1)	18 (56.3)	0.228
Internet	106 (27.0)	13 (12.3)	35 (32.1)	59 (55.7)	**0.048**
How many days ago last sex					**0.056**
Today	106 (27.0)	6 (5.7)	27 (25.5)	73 (68.9)	
1–2 days ago	135 (34.4)	8 (5.9)	39 (29.9)	88 (65.2)	
3–6 days ago	66 (16.8)	11 (16.7)	14 (21.1)	41 (62.1)	
7 or more days ago	86 (21.9)	6 (7.1)	31 (35.3)	49 (57.6)	
Condom used with last paying sex partner									
No	35 (8.9)	0 (0)	12 (34.3)	23 (65.7)	0.169
Yes	357 (91.1)	31 (8.7)	98 (27.5)	228 (63.9)	
Median (IQR) payment from last client, in Kenyan Shillings	700 (400-1500)	1000 (500–1500)	1000 (500–1500)	550 (350–1200)	**0.006**
General health related									
Taken antibiotics in past 30 days									
No	343 (87.5)	26 (7.6)	100 (29.2)	217 (63.3)	0.609
Yes	49 (12.5)	4 (8.2)	11 (22.5)	34 (69.4)	
Current HIV PrEP use (among those HIV negative, who heard of PrEP)									
No	150 (51.5)	14 (9.3)	53 (35.3)	83 (55.3)	0.442
Yes	141 (48.5)	14 (9.9)	40 (28.4)	87 (61.7)	
Hormonal birth control use									
None	127 (32.3)	17 (13.4)	35 (27.6)	75 (59.1)	**0.002**
Oral contraceptives	28 (7.1)	2 (7.1)	12 (42.9)	14 (50.0)	
Injection	110 (28.0)	7 (6.4)	39 (35.5)	64 (58.2)	
Implant	128 (32.6)	5 (3.9)	25 (19.5)	98 (76.6)	
STI and HIV Infections									
Nugent score									
0-3	174 (44.4)	30 (17.2)	98 (56.3)	46 (26.4)	**<0.001**
4-6	51 (13.0)		7 (13.7)	44 (86.3)	
7-10 (BV)	167 (42.6)		6 (3.6)	161 (96.4)	
STI									
Negative	309 (78.6)	30 (9.7)	98 (31.7)	181 (58.6)	**<0.001**
Positive (composite)	84 (21.4)	1 (1.2)	13 (15.5)	70 (83.3)	**0.005**
*C. trachomatis*	49 (12.5)	1 (2.0)	6 (12.2)	42 (85.7)	0.156
*N. gonorrhoeae*	20 (5.1)	0 (0.0)	3 (15.0)	17 (85.0)	0.073
*T. vaginalis*	29 (7.4)	0 (0.0)	5 (17.2)	24 (82.8)	
HIV status									
Negative	297 (75.8)	29 (9.8)	94 (31.6)	174 (58.6)	**<0.001**
Positive	95 (24.2)	2 (2.1)	17 (17.9)	76 (80.0)	
Among HIV positive participants									
Not taking antiretrovirals	16 (16.8)	1 (6.3)	4 (25.0)	11 (68.8)	0.205
Taking antiretrovirals	79 (83.2)	1 (1.3)	13 (16.5)	65 (82.3)	
HSV2 serostatus									
Negative	146 (37.4)	23 (15.8)	52 (35.6)	71 (48.6)	**<0.001**
Positive	244 (62.6)	8 (3.3)	58 (23.8)	178 (73.0)	

^1^CST was available for 395 participants with >2500 sequence reads; 2 participants with CST-II (n=1) and CST-V (n=1) were excluded.

^2^Includes n=14 participants with income >25,000 KSH.

^3^Locations of meeting clients that were not analyzed due to sparsity: escort service (n=6), market (n=3), mtatu or boda stand (n=7).

The bold values are p-values statistically significant at p<0.05.

### 3.3 Menstrual hygiene management and factors associated with use of sub-optimal materials

Overall, 97.8% (n=398/407) of participants reported menstruating in the past 6 weeks (**Table 2**). Nearly all (95.0%) women reported using disposable pads to manage menses, with a minority (5.3%) reporting reusable pads. Use of sub-optimal materials was common, with 42% reporting using any of the following: cotton wool (34.0%), tissues (14.1%), or cloth (8.1%). Difficulty accessing water was reported more frequently during sex work (23.8%) than at home (16.2%). Women reported they carried their own soap when doing sex work (57.5%) or that someone else provided soap (67.1%). Having difficulty for personal privacy was experienced by 12.5% of women while at home and by 18.7% during sex work. Over half of participants (57.0%) reported having fewer clients during menses compared to when they are not having menses. Overall, 56.0% of women wiped inside their vagina more frequently during menses than not during menses (on average 2.5 times per day vs. 1.9 times per day, p<0.001 sign rank test). The majority (92.9%) reported wiping inside the vagina with a cloth, tissue, or cotton to remove fluids between clients: always (76.9%), often (13.5%), sometimes (2.5%), never (7.1%).

**Table 2:** Distribution of menstrual hygiene management practices and conditions by community state type.

Variables	Total N=39 n (%)	CST-I, N = 31 n (%)	CST-III, N = 111 n (%)	CST-IV, N = 251 n (%)	p-value
Material used at last period (not mutually exclusive)^1^					
Disposable pad					
No	20 (5.2)	1 (5.0)	0 (0)	19 (95.0)	**0.002**
Part or all of period	365 (94.8)	29 (7.9)	109 (29.9)	227 (62.2)	
Cotton wool					
No	253 (65.7)	23 (9.1)	81 (32.0)	149 (58.9)	**0.018**
Part or all of period^2^	132 (34.3)	7 (5.3)	28 (21.2)	97 (73.5)	
Tissues					
No	332 (86.5)	29 (8.7)	99 (29.8)	204 (61.5)	**0.042**
Part or all of period^3^	52 (13.5)	1 (1.9)	10 (19.3)	41 (78.9)	
Cloth					
No	354 (92.4)	29 (8.2)	99 (28.0)	226 (63.8)	0.643
Part or all of period	29 (7.6)	1 (3.5)	10 (34.5)	18 (62.1)	
Any sub-optimal material for menses (cotton wool, tissues, cloth)					
No	225 (58.4)	22 (9.8)	77 (34.2)	126 (56.0)	**0.001**
Yes	160 (41.6)	8 (5.0)	32 (20.0)	120 (75.0)	
Characteristics of most recent period					
Median (IQR) days of bleeding at last period	3 (3 – 4)	3 (3 – 4)	3 (3 – 4)	3 (3 – 3)	0.352
Menstrual flow					
Normal	335 (87.0)	26 (7.8)	97 (29.0)	212 (63.3)	0.634
Light	26 (6.8)	1 (3.9)	8 (30.8)	17 (65.4)	
Heavy	24 (6.2)	3 (12.5)	4 (16.7)	17 (70.8)	
Median (IQR) days since last menstrual period	15 (7 – 23)	16 (10 – 25)	14 (8 – 27)	12 (6 – 22)	0.174
Estimated phase of menstrual cycle					
Menstrual (day 0-5)	19 (4.8)	0 (0)	6 (31.6)	13 (68.4)	0.479
Follicular (day 6-11)	86 (21.9)	7 (8.1)	21 (24.4)	58 (67.4)	
Ovulatory (day 12-16)	80 (20.4)	3 (3.8)	20 (25.0)	57 (71.3)	
Luteal (day 17-42)	180 (45.8)	19 (10.6)	56 (31.1)	105 (58.3)	
Uncertain	28 (7.1)	2 (7.1)	8 (28.6)	18 (64.3)	
Difficulty accessing water and/or privacy at home and/or sex work					
Difficulty accessing water at home					
No	329 (83.7)	30 (9.1)	95 (28.9)	204 (62.0)	**0.063**
Yes	64 (16.3)	1 (1.6.)	16 (25.0)	47 (73.4)	
Difficulty accessing water for cleaning during menses when doing sex work					
No	299 (76.1)	28 (9.4)	94 (31.4)	177 (59.2)	**0.002**
Yes	94 (23.9)	3 (3.2)	17 (18.1)	74 (78.7)	
Difficulty with personal privacy at home					
No	345 (87.8)	29 (8.4)	96 (27.8)	220 (63.8)	0.607
Yes	48 (12.2)	2 (4.2)	15 (31.2)	31 (64.5)	
Any difficulty with privacy for cleaning during menses when doing sex work					
No	318 (80.9)	28 (8.8)	98 (30.8)	192 (60.4)	**0.012**
Yes	75 (19.1)	3 (4.0)	13 (17.3)	59 (78.7)	
Menstrual hygiene management					
Adequate materials, water, and privacy	172 (44.7)	18 (10.5)	59 (34.3)	95 (55.2)	**0.003**
Adequate materials, difficulty accessing water and/or privacy	53 (13.8)	4 (7.5)	18 (34.0)	31 (58.5)	
Sub-optimal materials (with or without difficulty accessing water and/or privacy)	160 (41.5)	8 (5.0)	32 (20.0)	120 (75.0)	
Source of soap when doing sex work (not mutually exclusive)					
I carry my own soap	224 (57.0)	13 (5.8)	61 (27.2)	150 (67.0)	0.147
The caretaker/manager at venue provides soap	66 (16.8)	6 (9.1)	14 (21.2)	46 (69.7)	0.147
Someone else provides soap	265 (67.4)	26 (9.8)	81 (30.6)	158 (59.6)	**0.022**
There is no soap available	2 (0.5)	0	0	2	
Intravaginal Practices					
How often do you use cloth, tissue paper, or cotton to wipe inside your vagina?					
Never	28 (7.1)	0 (0)	8 (28.6)	20 (71.4)	0.308
Ever	365 (92.9)	31 (8.5)	103 (28.2)	231 (63.3)	
How often do you put something inside your vagina to keep it dry during menses?					
Never	263 (67.1)	20 (7.6)	71 (27.0)	172 (65.4)	0.633
Ever	129 (32.9)	11 (8.5)	40 (31.0)	78 (60.5)	
Median (IQR) number of times per day wipe inside vagina:					
When having menses	2 (2-3)	2 (2-3)	2 (2-3)	2 (2-3)	0.948
When not having menses	2 (2-2)	2 (2-2)	2 (2-2)	2 (2-2)	0.233
Wipe inside vagina more times during menses than when not having menses					
No	176 (44.8)	16 (9.1)	44 (25.0)	116 (65.9)	0.371
Yes	217 (55.2)	15 (6.9)	67 (30.9)	135 (62.2)	
Ever use commercial (i.e., store bought) douche in the past 6 months					
No	362 (92.4)	27 (7.5)	101 (27.9)	234 (64.6)	0.285
Yes	30 (7.6)	4 (13.3)	10 (33.3)	16 (53.3)	

^1^Of N = 393 women with CST-I, -III, -IV, and >2500 sequence reads, these responses restricted to n=385 (98.0%) experiencing menses in past 6 weeks.

^2^Includes n=11 participants who reported using cotton balls to manage for entire period, all with CST-IV.

^3^Includes n=4 participants who reported using tissues to manage for entire period, all with CST-IV.

The bold values are p-values statistically significant at p<0.05.

**Figure 2:**
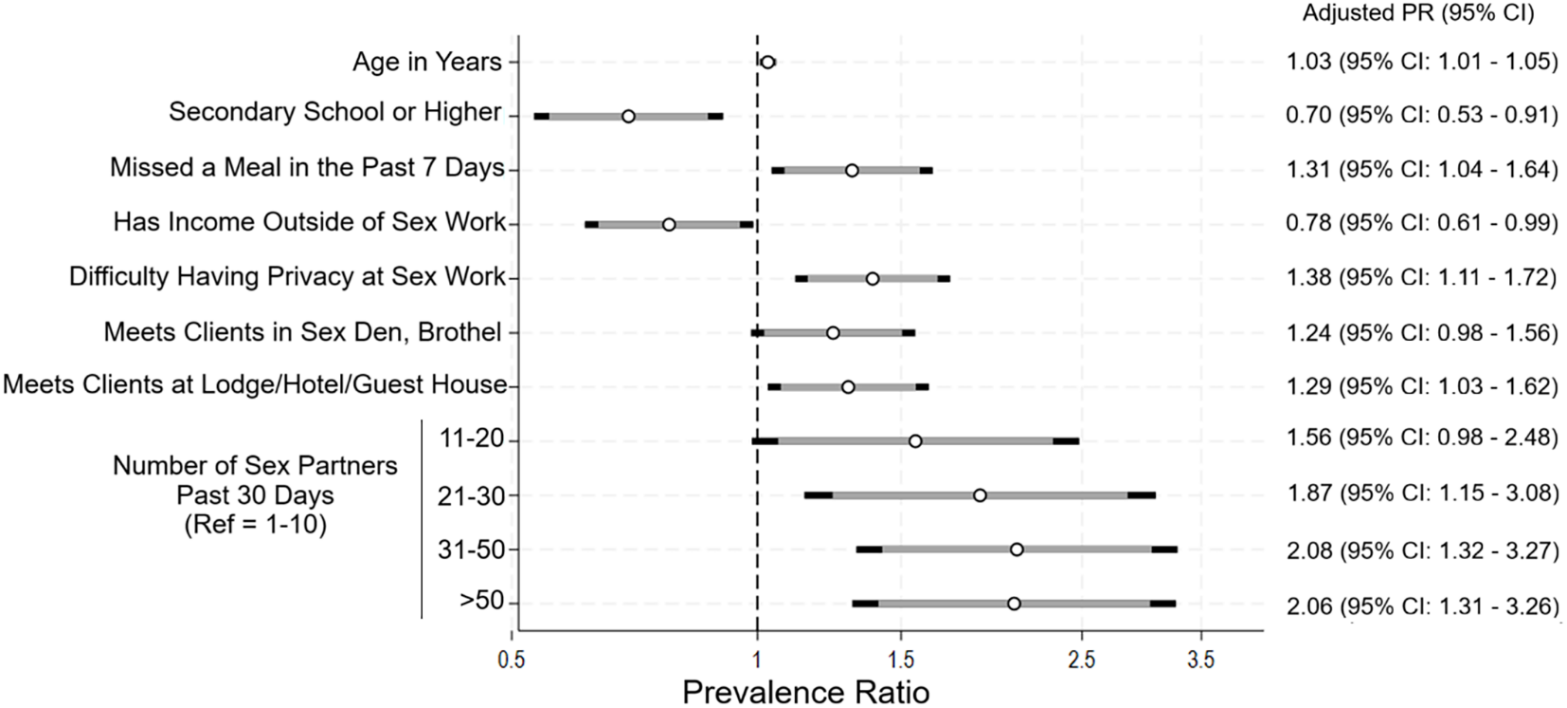
Results of multivariable regression: factors associated with sub-optimal menstrual materials, N = 398. The coefficient plot shows the prevalence ratio (open circle) and 95% confidence interval (grey horizontal bar 90% CI; black horizontal ends 95% CI) for each variable associated with using sub-optimal menstrual materials in multivariable regression. All variables presented are simultaneously adjusted.

In multivariable regression, factors associated with using sub-optimal MM included older age, lower educational attainment, and indicators of economic strain (missing a meal due to lack of money, not having income outside of sex work) (**Figure 2**; **Supplementary Table 1**). Difficulty having privacy at sex work, meeting clients at specific types of sex work venues (sex den/brothel, lodge/hotel/guest house), and having 20 or more clients in the past 30 days were also associated with increased likelihood of using sub-optimal MM.

### 3.4 Characteristics of the vaginal microbiome

**Figure 3:**
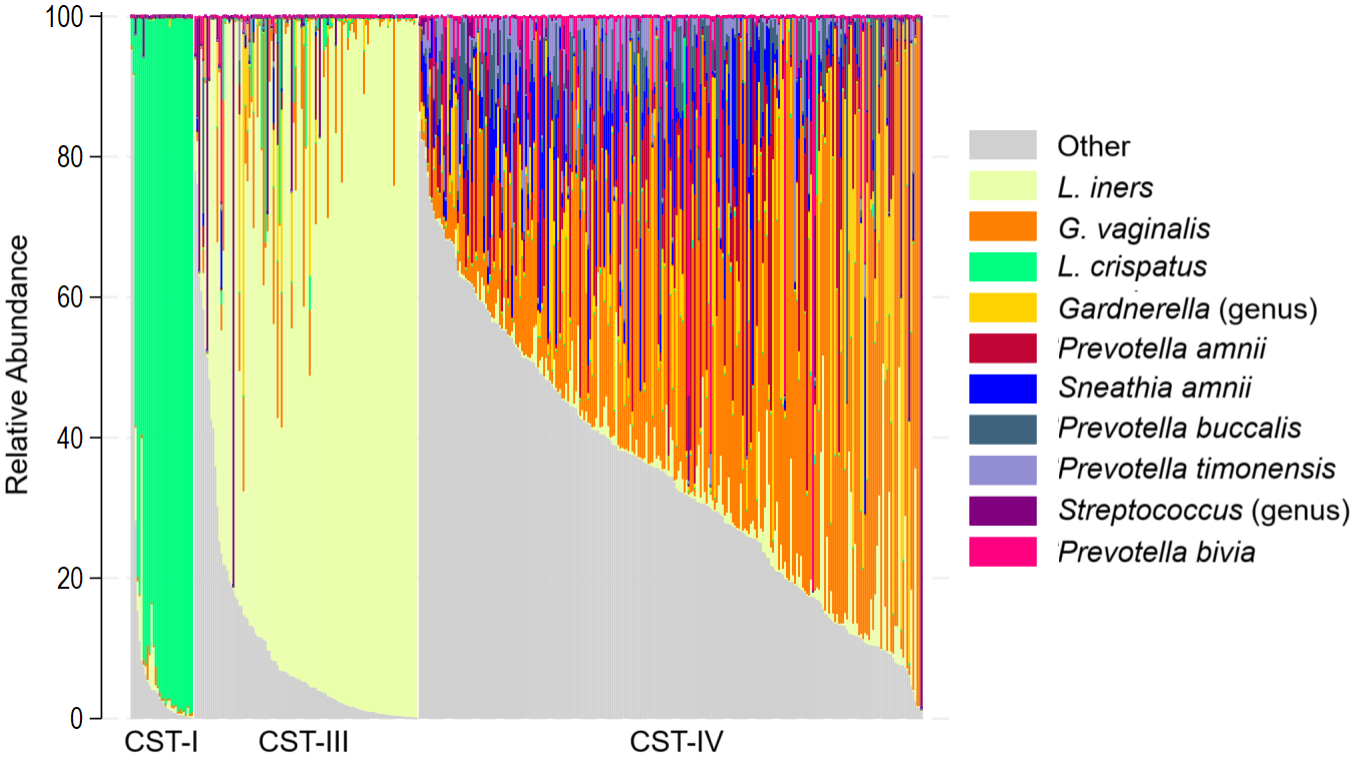
Stacked bar chart showing relative abundance of 10 taxa with highest mean relative abundance by community state type for each participant. The relative abundance of the 10 taxa with the highest mean relative abundance is shown (y-axis), with individual subjects represented by individual bars, sorted by CST (x-axis).

Among the 395 participants included in VMB analysis, CST-IV (diverse) was the most common, identified in 251 (63.4%) women, primarily of sub-type IV-B (n=243). CST-III (*L. iners* dominated) was the next most common (28.3%), followed by CST-I (7.8%, *L, crispatus* dominated). The top 10 taxa with the highest relative abundance (**Figure 3**) accounted for 73.3% of sequence reads on average.

#### 3.4.1 Factors associated with community state type

The distribution of CST varied by numerous factors, and in relation to the primary exposures of interest, CST-IV was more common among women who reported: sub-optimal MM, difficulty accessing water at home and when doing sex work, and having difficulty with privacy for menses during sex work. Among participants with optimal CST-I, no BV was detected (vs. 6.8% and 63.9% for participants with CST-III and CST-IV respectively), just one case of STI (3.2% vs. 12.5% and 27.9% for participants with CST-III and CST-IV, respectively), and 2 cases of HIV (6.5% vs. 15.2% and 30.4% for participants with CST-III and CST-IV, respectively). The association of menstrual management with CST was specific to sub-optimal materials: the distribution of CST was similar for women with adequate menstrual management material, water and privacy access, and for women with adequate material but difficulty accessing water and/or privacy; increased prevalence of CST-IV was observed only for women reporting sub-optimal menstrual materials (**Table 1**). Difficulty accessing water and privacy were nearly collinear (**Supplementary Figure 1**), and could not be examined separately.

Participants with sub-optimal MM were more likely to have CST-IV than CST-I (prevalence ratio [PR] = 2.62; 95% CI: 1.12 – 6.12) (**Table 3**), though in multivariable adjusted analyses, CST was no longer associated with sub-optimal MM: the association was attenuated and non-significant in the presence of age, educational attainment, amount paid at last sexual encounter, number of sex partners, and HSV-2. CST-III and CST-IV were more likely with older age, though associations were of marginal significance. Receiving median or greater payment at last sex act was inversely associated with having CST-IV, also of marginal significance. There was increased likelihood of CST-III and CST-IV with hormonal contraceptive use, but this reached statistical significance (p<0.05) only for injectable contraceptive use and CST-III vs. CST-I (aPR = 3.12) and implant contraceptive use and CST-IV (aPR = 4.11). Both CST-III and CST-IV were more likely for those testing positive for HSV-2 or an STI, though this was significant at p<0.05 level only for CST-IV (aPR = 4.82 for HSV-2 and aPR = 12.7 for STI).

**Table 3:** Results of multinomial regression: factors associated with vaginal community state type.

	Prevalence ratio (95% CI)		Adjusted prevalence ratio, N = 382 (95% CI)	
Variables	CST-III vs CST-I	CST-IV vs CST-I	CST-III vs CST-I	CST-IV vs CST-I
Sub-optimal materials (vs. adequate MM)	1.14 (0.46 – 2.84)	2.62 (1.12 – 6.12)	0.68 (0.23 – 2.04)	1.32 (0.46 – 3.82)
Age in years, continuous	1.13 (1.02 – 1.24)	1.19 (1.08 – 1.30)	1.07 (0.96 – 1.10)	1.08 (0.97 – 1.19)
Educational attainment: Secondary high school or more	0.33 (0.14 – 0.78)	0.29 (0.13 – 0.63)		
Received 700 KSH or more at last sexual act	0.47 (0.19 – 1.15)	0.29 (0.13 – 0.68)	0.58 (0.22 – 1.51)	0.43 (0.17 – 1.10)
Has a non-paying boyfriend or husband	1.33 (0.53 – 3.36)	0.67 (0.29 – 1.56)		
Hormonal contraceptive use				
None	ref	ref	ref	ref
Oral contraceptive pills	2.91 (0.58 -14.5)	1.59 (0.33 – 7.66)	2.75 (0.48 – 15.7)	1.69 (0.31 – 9.36)
Injection	2.71 (1.00 – 7.30)	2.07 (0.81 – 5.32)	3.02 (1.01 – 9.00)	2.30 (0.78 – 6.80)
Implant	2.43 (0.79 – (7.46)	4.44 (1.57 - 12.6)	2.13 (0.67 – 6.81)	4.03 (1.29 – 12.6)
Number of sex partners in past 30 days				
0-10	ref	ref	ref	ref
11-30	1.43 (0.55 – 3.74)	2.13 (0.85 – 5.32)	1.79 (0.63 – 5.09)	2.61 (0.91 – 7.50)
Greater than 31	1.39 (0.50 – 3.89)	3.03 (1.15 – 8.00)	1.70 (0.57 – 5.04)	2.84 (0.99 – 8.17)
HIV positive	2.62 (0.57 – 12.1)	6.33 (1.47 – 27.3)		
HSV-2 seropositive	3.21 (1.32 – 7.80)	7.21 (3.08 – 16.9)	2.71 (0.92 – 7.97)	4.65 (1.60 – 13.4)
STI positive	3.98 (0.50 – 31.8)	11.6 (1.55 – 86.9)	3.95 (0.49 – 31.8)	13.0 (1.65 – 102)
Meet sex partners at sex den or brothel	1.69 (0.53 – 6.27)	3.22 (1.09 – 9.51)		
Meet sex partners via Internet	0.61 (0.27 – 1.39)	0.43 (0.20 – 0.92)		
Difficulty accessing water at home	5.05 (0.64 – 39.8)	6.91 (0.92 – 52.1)		
Difficulty accessing water at sex work	1.69 (0.46 – 6.19)	3.90 (1.15 – 13.3)		
Difficulty accessing privacy at sex work	1.24 (0.33 – 4.66)	2.87 (0.84 – 9.79)		
Days since last vaginal sex				
Today	ref	ref		
1–2 days	1.08 (0.34 – 3.48)	0.90 (0.30 – 2.73)		
3–6 days	0.28 (0.09 – 0.93)	0.31 (0.11 – 0.89)		
7 days or more	1.15 (0.33 – 3.99)	0.67 (0.20 – 2.21)		

#### 3.4.2 Factors associated with alpha diversity

**Figure 4:**
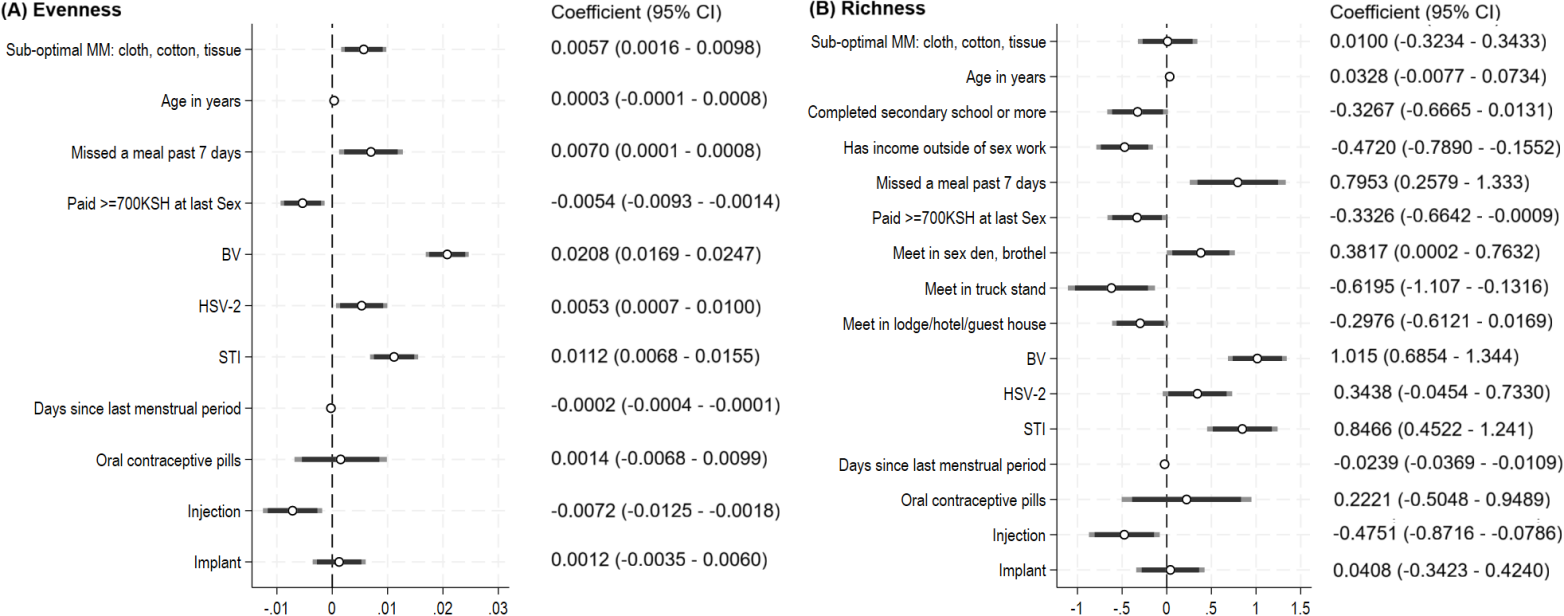
Results of multivariable regression: factors associated with alpha diversity metrics. The plots summarize the results of multivariable linear regression for **(A)** Evenness (N = 383), and **(B)** Richness (N = 383). Variables are listed on the y-axis. Coefficients (central dot) and 95% Confidence Interval (black horizontal bar with 90% and 95% CIs) are summarized graphically, with values on the x-axis. The reference for oral contraceptive pills, injection contraceptive, and implant contraceptive is no hormonal contraception. “Missed a meal in past 7 days” refers to having missed a meal in the past 7 days due to not having enough money. “Meet in sex den, brothel” refers to meeting clients in sex dens or brothels, which are premises dedicated to providing sex as a purchased service.

Alpha diversity metrics were lowest for individuals who were negative for BV, STIs, HSV-2, and HIV (**Supplementary Figure 2**). In multivariable linear regression, sub-optimal MM was positively associated with all alpha diversity metrics, with p<0.05 for evenness, and with marginal significance (0.05<p<0.10) for the other measures (**Figure 4**). Additionally, alpha diversity increased with BV, STI and HSV-2 infection, meeting sex partners in sex dens or brothels, or having missed a meal in the past 7 days due to lack of money and were decreased with use of injectable hormonal contraception. For richness and evenness metrics, being paid 700 KES (~$5.50 USD) or more at the last sex act was associated with lower alpha diversity as was increasing days since last menstrual period. Most covariates and associations were similar across alpha diversity metrics, given that the Spearman correlations ranged from 0.783 to 0.997 among measures.

#### 3.4.3 Results of non-targeted analyses: taxa associations with use of sub-optimal menstrual materials

Among participants with no infections (BV, STI, HSV-2, HIV), non-targeted analyses did not identify any taxa differing between those with sub-optimal MM compared to those with adequate MM. Among 386 participants with VMB read count >2500 and having menstruated within the past 6 weeks, adjusting for infection with BV, STI, HSV-2, and HIV, 3 taxa with the highest probability of selection were *Peptococcus* spp., *Mobiluncus curtisii*, and *Prevotella bivia* (**Table 4**).

**Table 4:** Results of lasso regression implemented with stability selection: mean relative abundance of taxa associated with using sub-optimal menstrual materials, coefficients from logistic regression.

Analysi sample	Mean relative abundance (SD)		Percent of bootstraps in which the taxa were selected at various p-values		
Among those with no infection	Sub-Optimal MM,N = 25	Adequate MM,N = 55	P<0.10	P<0.15	P<0.20
*No significant taxa detected*					

All observations Adjusted for BV, STI, HSV-2, HIV	Sub-Optimal MM, N = 161	Adequate MM N=225	P<0.10	P<0.15	P<0.20
*Peptococcus* spp.	0.033 (0.172)	0.022 (0.131)	81	81	85
*Mobiluncus curtisii*	0.026 (0.164)	0.009 (0.040)	74	79	84
*Prevotella bivia*	2.53 (7.37)	1.55 (7.98)	75	77	81
*Gemella asaccharolytica*	0.312 (0.660)	0.235 (0.762)	64	65	67
*Aerococcus christensenii*	0.305 (0.771)	0.488 (1.32)	62	66	69

SD, Standard Deviation.

**Figure 5:**
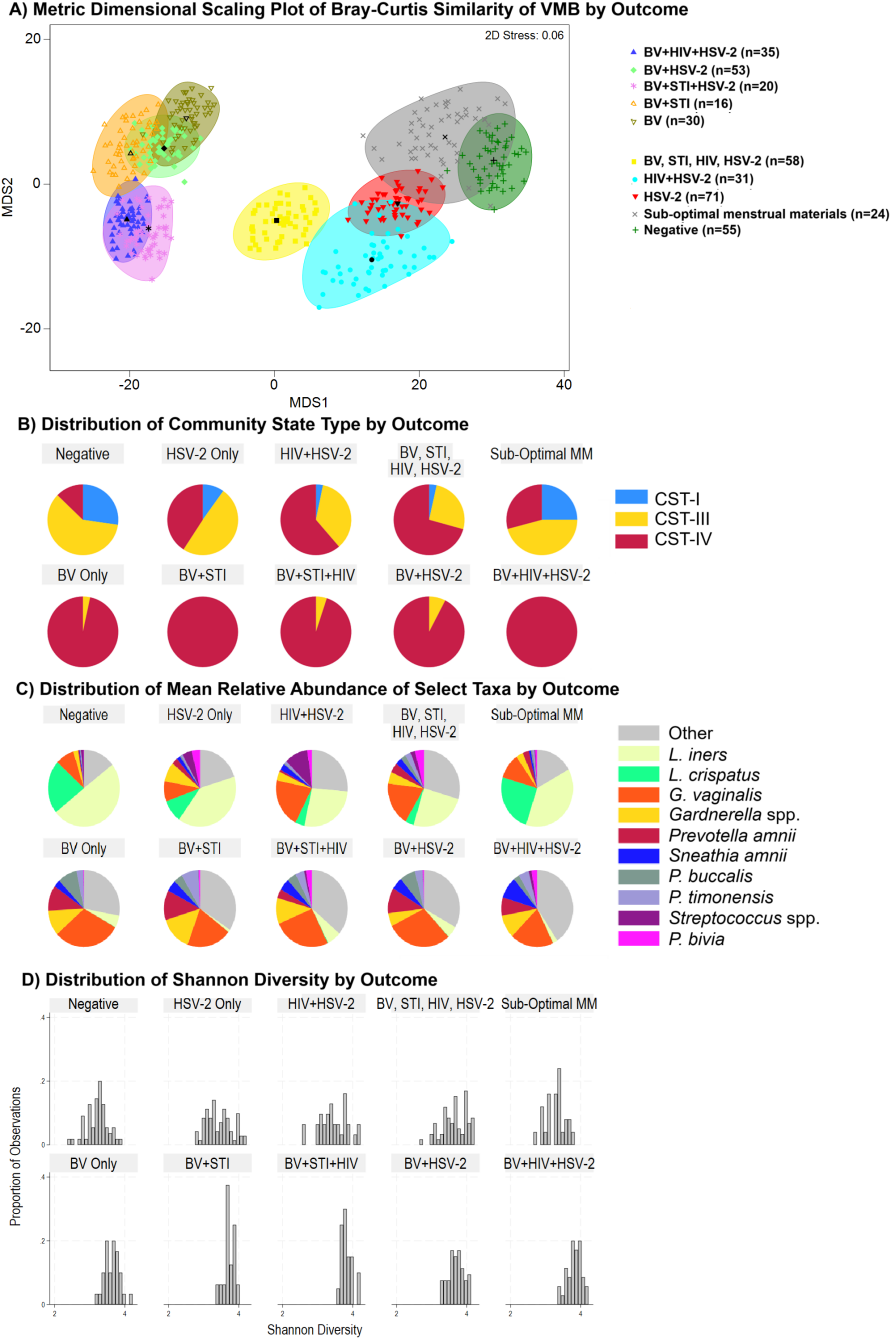
Metric dimensional scaling plot of group centroids and distributions of CST, taxa relative abundance, and Shannon diversity by outcomes. Caption: Panel **(A)** Non-metric dimensional scaling plot for each of the outcome states for Bacterial vaginosis (BV) or sexually transmitted infection (STI). The ten different colors represent the states for BV, STIs, HIV, HSV-2, and sub-optimal menstrual materials. Sub-optimal menstrual materials refer to use of cotton balls, tissue, or cloth to manage menses, in the absence of other infections. STI is a composite of infection with any of *C. trachomatis, N. gonorrhoeae, T. vaginalis.* Each colored mark indicates one of 100 bootstraps of the dataset. The matching shaded area represents the 95% coverage. The black symbol at the center of each colored shape represents the average centroid of the 100 bootstraps. Panel **(B)** distribution of Community State Type (CST); Panel **(C)** taxa relative abundance; Panel **(D)** Shannon diversity.

Analysis of similarity via the Bray-Curtis resemblance matrix demonstrated that the VMB composition grouped into two domains (**Figure 5**, Panel A): one in which participants had BV alone or in combination with other infections (left), and one in which participants did not have BV (right). One infection category (yellow, center, labelled “BV, STI, HIV, HSV”) included outcomes that were too sparse to analyze on their own: n=14 STI only, n=12 STI + HSV, n=12 STI+HIV+HSV, n=9 BV+STI+HIV+HSV, n=5 HIV only, n=1 BV+HSV, n=1 BV+STI+HIV. The participants who had BV (**Figure 5**, Panel A, left side cluster) – whether alone or in combination with other infections – were largely CST-IV dominant (**Figure 5**, Panel B), while those without BV (**Figure 5**, Panel A, right side cluster) had more prevalent CST-III and CST-I (**Figure 5**, Panel B). The distributions of the relative abundance of taxa (**Figure 5**, Panel C) and Shannon diversity (**Figure 5**, Panels D) were in keeping with these CST differences, with higher alpha diversity among outcomes that included BV and greater proportion of CST-IV.

The global vaginal microbiome composition of women who tested negative for infections and had sub-optimal vs. adequate MM was not statistically significantly different (**Supplementary Table 2**), and this is reflected in the close positioning of centroids and coverage areas shown for these two groups in **Figure 5**, Panel A (dark green and grey areas). The relative abundance and presence/absence of taxa for those with sub-optimal MM compared to those with adequate MM and no infections is summarized in **Supplementary Table 3**.

## 4 Discussion

We measured the VMB and association with menstrual practices among 407 women who rely on sex for economic livelihood in western Kenya. A minority of women had optimal, *L. crispatus* dominated CSTs. Sub-optimal MM – using cotton balls, tissues, or cloth to manage menses - was common and more likely among women with indicators of greater economic strain. Sub-optimal MM was associated with non-optimal VMB composition in various analyses.

We collected a wide range measures of menstrual practices: materials used, difficulty accessing water or privacy (separately at home and at sex work), and source of soap. Use of sub-optimal materials was prevalent, and many women experienced difficulty accessing water and privacy, with higher frequencies of challenges reported when doing sex work. While MM factors were associated with vaginal CST in crude analyses, none were associated in multivariable analyses. Age, educational status, payment for sex work, and number of sex partners may arguably be in the causal pathway (i.e., leading to use of sub-optimal MM and working in more challenging settings), and indeed increasing age, lower educational attainment, and indicators of lower socioeconomic status were associated with increased likelihood of sub-optimal MM. This finding aligns with meta-analyses showing that higher educational attainment and economic status are associated with increased likelihood of adequate MHM (Anbesu and Asgedom, 2023). Thus because sub-optimal MM could have a shared socioeconomic component with nearly all of the variables associated with vaginal CST, we opted to present the best fitting model, and explain which covariates led to attenuation of the association between MM and CST.

Although menstrual hygiene management factors were not associated with CST, use of sub-optimal menstrual materials was associated with increased alpha diversity metrics, controlling for measures of economic strain, STIs, HSV-2 and hormonal contraceptive use. Additionally, non-targeted analyses identified non-optimal taxa discriminating those using sub-optimal MM from those using adequate MM, adjusted for reproductive tract infections. While we did not observe statistically significant global difference between the vaginal communities of women with and without adequate MM, those with sub-optimal MM had lower relative abundance of multiple *Lactobacilli* species.

Among women who tested negative for BV, STIs, HIV and HSV-2, non-targeted analyses did not identify any taxa that discriminated those who had sub-optimal and adequate MM. In the non-targeted analysis making use of the entire sample and adjusting for other infections, we identified three taxa with greater relative abundance among women with sub-optimal MM that have all been associated with BV, with *Mobiluncus curtisii* and *Prevotella bivia* being repeatedly identified in BV-related biofilm formation (Pybus and Onderdonk, 1999; Johnston et al., 2023). None were highly abundant, but the analytic approach prioritizes discrimination (i.e., separating one group from others). Together, these analyses suggest that even after adjusting for BV, HIV, STIs, or HSV-2, sub-optimal MM was associated with greater relative abundance of non-optimal bacteria. The results of non-targeted analyses are exploratory; replication is necessary and specific taxa should be interrogated in relation to sub-optimal MM in other studies.

Non-menstrual hygiene factors associated with increased alpha diversity and non-optimal CST were largely in keeping with the literature: sexual exposures, reproductive tract infections (HSV-2, BV, STIs) and indicators of lower socioeconomic status (missing a meal due to lack of money, lower educational attainment, not having income outside of sex work) (van de Wijgert JH et al., 2014; McKinnon et al., 2019; Moosa et al., 2020; Morsli et al., 2024). Regarding the association with meeting clients in sex dens or brothels, differences in VMB composition may stem from characteristics of men soliciting sex at different venues (e.g., their prevalence of infection, circumcision status, number and type of other sex partners) or residual confounding by socioeconomic status: women meeting clients in sex dens or brothels were less likely to be paid the median or higher at the last sex act (33.9%) as compared to women meeting clients in their homes (67.9%), bars or clubs (54.1%), lodges/hotels/guest houses (53.6%), at parties/social gatherings (63.6%), or via the internet (73.2%). Compared to those with no hormonal contraceptive use, those with implant and injection contraceptive use had increased likelihood of non-optimal VMB and increased alpha diversity; this is in contrast to meta-analysis showing protective association with BV (Vodstrcil et al., 2013). Our observation may stem from residual confounding, or the low frequency of CST-I in our sample. While antibiotic use can affect the VMB, we did not find such association, which may be due to misreporting or lack of information on drug class, dose or duration. Vaginal douching was not associated with VMB in our cohort, which is in contrast to the literature (Ness et al., 2002), and may be due to the way in which we asked the question. Estimated menstrual cycle phase also was not associated with VMB and may have been due to the indirect method of estimation (Wideman et al., 2013).

In this cohort of women reporting reliance on sex for economic livelihood, just 7.9% had an optimal, *L. crispatus* dominated VMB composition. This prevalence of CST-I is similar to that observed among our study of a community-based sample of women median age 23 in Kisumu, who had 10.8% CST-I, 40.6% CST-III, and 46.5% CST-IV (Mehta et al., 2020), despite much lower prevalences of HIV, BV, HSV-2 and sexual exposures. The similar CST distribution of a community-based cohort of women and a cohort of women with very high sexual exposures suggests there is little reversion from non-optimal states (CST-III and CST-IV) once acquired. Indeed, BV is a chronic state for many women with 50-65% recurrence within 6–12 months of antibiotic treatment (Bradshaw et al., 2006). Although recurrence can be reduced with male sex partner treatment (Vodstrcil et al., 2025), this is unlikely to be a feasible option for women engaged in sex work. Therefore, identifying non-antibiotic interventions that help maintain optimal VMB or mitigate non-optimal transitions will be critical for primary and secondary prevention.

### 4.1 Limitations

A small number of participants had CST-I, reducing power in this aspect of the analysis, and thus few factors associated with CST reached statistical significance at the p<0.05 level. In our non-targeted analysis, we observed that the taxa selected were not limited to the most common or abundant; however, taxa with low relative abundance may have lower variation in sub-samples of stability selection, smaller effect size, and therefore lower selection frequency across sub-samples. We measured menstrual materials used at most recent menses for ease of recall. Though our findings were in keeping with measures of menstrual management in the region (Phillips-Howard et al., 2015; UNFPA, 2025), it is possible this is not representative of women’s ongoing or typical practices. Nevertheless, the directions of the associations observed were in keeping with prior literature or had rational biological basis. Socioeconomic indicators were statistically significant in our analyses and associations are supported in the literature; however, we lacked a standardized index that would support direct comparability of socioeconomic status to other settings. Despite the high frequency of reported condom use, STIs and HIV were common, indicating that use may be over-reported or insufficiently consistent, reflecting an interventional gap. Ulcerative and non-ulcerative STIs have bidirectional effects on the VMB and in this cross-sectional analysis we do not know temporality; however, with longitudinal follow-up of this cohort and repeated STI testing, subsequent analyses will enable us to estimate changes in the VMB in response to incident STI and subsequent treatment. Our nested analysis within a cluster randomized study among adolescent girls found that sub-optimal MM was associated with increased likelihood of non-optimal VMB (Mehta et al., 2021) and that provision of menstrual cups had beneficial effects for the VMB (Mehta SD et al., 2023). Other observational studies have similarly found beneficial association between menstrual cups and VMB (Lebeer et al., 2023; Haahr et al., 2025). To test the generalizability of this finding, we intentionally expanded study of MM and VMB to a population with very high rates of sexual exposures and reproductive tract infections. Our findings add to the limited knowledge of menstrual materials and relation to VMB and reproductive tract health, and similar studies should be undertaken in a variety of settings to better identify avenues for optimizing menstrual health management and reproductive health benefit.

## 5 Conclusions

Interventions to mitigate non-optimal VMB are needed, especially among women with excess risk for HIV and STIs. Reusable menstrual cups, which last for up to 10 years, may address the economic factors driving use of sub-optimal menstrual materials, with subsequent impact on VMB, and factors associated with non-optimal perturbations. We are investigating soft disc-shaped menstrual cups that can be worn during sex as a possible intervention, which may be especially suitable in supporting economic livelihood and vaginal tract health, by reducing direct perturbation and unsafe menstrual practices.

## Data Availability

The datasets presented in this study can be found in online repositories. The names of the repository/repositories and accession number(s) can be found below: https://www.ncbi.nlm.nih.gov/, PRJNA1279642.

## Supplementary material

Supplementary Figure 1 Proportional Venn Diagram Showing the Overlap of Using Sub-Optimal Menstrual Materials (Cotton Balls, Tissues, Cloth), Difficulty Accessing Water at Home and/or at Sex Work, and Difficulty Accessing Privacy at Home and/or at Sex Work.

Supplementary Figure 2 Distribution of alpha diversity metrics over infection and outcomes.

Supplementary Methods DNA Extraction and Sequencing.

Supplemental Results Akaike Information Criterion aided selection of multivariable models.

Supplemental Results Screening and Enrollment Flow, POWWeR Health Study.
