# Supplementary material for: Sub-optimal menstrual materials and vaginal microbiome disruption in women relying on sex for livelihood
Source: Front Cell Infect Microbiol. 2026 Jan 12;15:1662237. doi: 10.3389/fcimb.2025.1662237 (PMC12833416; doi:10.3389/fcimb.2025.1662237)
Supplement: Supplementary Figure 1 — Proportional Venn Diagram Showing the Overlap of Using Sub-Optimal Menstrual Materials (Cotton Balls, Tissues, Cloth), Difficulty Accessing Water at Home and/or at Sex Work, and Difficulty Accessing Privacy at Home and/or at Sex Work. [file DataSheet1.docx]

Supplementary Material

# Supplementary Methods - DNA Extraction and Sequencing

Genomic DNA was extracted from vaginal swabs using bead-beating and automated purification using a chemagic DNA Stool 200 Kit H96 (Revvity, Hamburg, Germany) implemented on a Chemagic 360 device. Swabs were initially placed in ZR BashingBead Lysis Tubes (0.1 & 0.5 mm) (S6012-50; Zymo Research) with 1 ml lysis buffer and 20 µl proteinase K (both from the chemagic DNA stool kit). Samples were subject to thermal mixing at 70°C for 10’ (device details), followed by bead-beating using a TissueLyzerII device (Qiagen) set at 30 Hz for 30”. A two-minute rest was provided in between two sequential bead-beating episodes. Subsequently, a five-minute incubation at 95°C was performed before final purification on the Chemagic360 instrument according to the manufacturer’s instructions.

Subsequently, genomic DNA was prepared for sequencing on a PacBio Revio sequencer using a two-stage PCR protocol, similar to that described for Illumina amplicon library preparation (Naqib et al. 2018), as described previously (Verma et al. 2025). In the first stage of PCR, DNA was PCR amplified with primers 27F and 1492R (TTTCTGTTGGTGCTGATATTGCAGRGTTYGATYMTGGCTCAG and ACTTGCCTGTCGCTCTATCTTCRGYTACCTTGTTACGACTT, respectively) using domain-level bacterial primers. The underlined regions, however, represent Oxford Nanopore ‘Universal sequences for tailing PCR primers’ linker sequences (Matsuo et al. 2021). In the second stage of PCR, amplicons from the first stage were used as template, and amplified with primers that contained PacBio Kinnex adapter sequences, combinatorial dual indices, and the ONT linker sequences at the 3’ ends of the primers (Kinnex_Fwd: **CTACACGACGCTCTTCCGATCT**NNNNNNNNNNTTTCTGTTGGTGCTGATATTGC and Kinnex_Rev: **AAGCAGTGGTATCAACGCAGAG**NNNNNNNNNNACTTGCCTGTCGCTCTATCTTC).

Kinnex adapters are bolded, combinatorial indices are indicated with “NNNNNNNNNN”, and ONT linkers are underlined. First stage PCR amplifications were performed in 10 microliter reactions in 96-well plates, using repliQa HiFi ToughMix (Quantabio). Genomic DNA input was two microliters per reaction, and primers were added at 300 nM final concentration. PCR conditions were 98°C for 2 minutes, followed by 24 cycles of 98°C for 10 sec, 50°C for 2 sec and 68°C for 3 sec. The second PCR amplification was performed in 10 microliter reactions in 96-well plates using repliQa HiFi ToughMix. Each well received a separate primer pair containing unique combinatorial indices. One microliter of PCR product from the first stage amplification was used as template for the 2nd stage, without cleanup. Two microliters of primer were used per reaction. Cycling conditions were 98°C for 2 minutes, followed by 8 cycles of 98°C for 10 sec, 60°C for 1 sec and 68°C for 2 sec. PCR products were pooled and cleaned using a 0.5X Ampure cleanup.

PCR products from the 2^nd^ stage of amplification were pooled and purified and subject to Kinnex library preparation and loading on a PacBio Revio instrument. Two-stage library preparation was performed at the Genomics and Microbiome Core Facility (GMCF) at Rush University and Kinnex library preparation and PacBio sequencing were performed at the DNA Services Facility at the Roy J. Carver Biotechnology Center at the University of Illinois at Urbana-Champaign.

Amplicon microbiome bioinformatics analysis was performed with the software package QIIME2 2023.2 (Bolyen et al. 2019). Raw sequence data were checked for quality using seqkit (Shen et al. 2016). Primer sequences were removed using the cutadapt algorithm (Martin 2011). The trimmed sequences were denoised using DADA2 (Callahan et al. 2016) via the q2‐dada2-ccs algorithm, and ASVs generated from DADA2 were classified using the Naive Bayes classifier (Bokulich et al. 2018). Alpha‐diversity metrics (observed features (Faith 1992), Shannon Index (Shannon 1948), Simpson’s Index (Simpson 1949), and Pielou’s Evenness (Pielou 1966)) and beta diversity metrics were calculated using q2‐diversity. Taxonomy was assigned to ASVs against multiple databases to find the best match and reported accordingly. SILVA 138 (Quast et al. 2012; Yilmaz et al. 2014), Refseq+ (O'Leary et al. 2016), RDP (Cole et al. 2014), and the GTDB r202 reference database (Parks et al. 2020; Parks et al. 2018) was utilized to generate annotations. The contaminant removal software, decontam (Davis et al. 2018), was employed to detect any contaminants based on the prevalence of the ASVs in the reagent negative blank controls using default parameters. Community state types (CSTs) were identified in a reference dataset using nearest centroid classification (*VA*gina*L* community state typ*E N*earest *C*entro*I*d cl*A*ssifier (VALENCIA)).

# Supplemental Results: Akaike Information Criterion aided selection of multivariable models

1. Model: Factors associated with poor menstrual materials

aic_model_selection poisson poormhm educ_dich calcage i.numsp30d q109_reco q59_5 q24 medianpaid q59_7 outside q11_reco

AIC Model

614.5591 poormhm educ_dich

610.6941 poormhm educ_dich calcage

604.0453 poormhm educ_dich calcage i.numsp30d

*600.344 poormhm educ_dich calcage i.numsp30d q109_reco

*600.4483 poormhm educ_dich calcage i.numsp30d q109_reco q59_5

*600.3265 poormhm educ_dich calcage i.numsp30d q109_reco q59_5 q24

601.699 poormhm educ_dich calcage i.numsp30d q109_reco q59_5 q24 medianpaid

*600.9448 poormhm educ_dich calcage i.numsp30d q109_reco q59_5 q24 medianpaid q59_7

***600.9139 poormhm educ_dich calcage i.numsp30d q109_reco q59_5 q24 medianpaid q59_7 outside

602.9117 poormhm educ_dich calcage i.numsp30d q109_reco q59_5 q24 medianpaid q59_7 outside q11_reco

*Comparing models with comparable AICs, the model at (***) is the most informative. In this model, medianpaid is not significant (p=0.238) and the crude association (PR=0.69) is attenuated to 0.83 (AdjPR). There is no evidence of confounding when removed from the model (change in any other coefficients >10%). Therefore remove medianpaid.

FINAL MODEL

poisson poormhm educ_dich calcage i.numsp30d q109_reco q59_5 q24 q59_7 outside , r ir

1. Model: Factors associated with community state type

aic_model_selection mlogit cst134 poormhm hsv sti calcage i.hormonal2 medianpaid numsp30d2 educ_dich q59_5 q109_reco q61_c dayslast q59_11 if read>2500

AIC Model

641.5297 cst134 poormhm

616.3931 cst134 poormhm hsv

598.1275 cst134 poormhm hsv sti

599.5016 cst134 poormhm hsv sti calcage

* 596.5008 cst134 poormhm hsv sti calcage i.hormonal2

* 596.0994 cst134 poormhm hsv sti calcage i.hormonal2 medianpaid

*** 596.1384 cst134 poormhm hsv sti calcage i.hormonal2 medianpaid numsp30d2

* 596.777 cst134 poormhm hsv sti calcage i.hormonal2 medianpaid numsp30d2 educ_dich

600.3052 cst134 poormhm hsv sti calcage i.hormonal2 medianpaid numsp30d2 educ_dich q59_5

599.1897 cst134 poormhm hsv sti calcage i.hormonal2 medianpaid numsp30d2 educ_dich q59_5 q109_reco

601.5039 cst134 poormhm hsv sti calcage i.hormonal2 medianpaid numsp30d2 educ_dich q59_5 q109_reco q61_c

606.0671 cst134 poormhm hsv sti calcage i.hormonal2 medianpaid numsp30d2 educ_dich q59_5 q109_reco q61_c dayslast

609.5731 cst134 poormhm hsv sti calcage i.hormonal2 medianpaid numsp30d2 educ_dich q59_5 q109_reco q61_c dayslast q59_11

*Comparing the models with comparable AIC: number of sex partners has marginally significant Wald p-values and a large effect size. Keep this as more informative model. Education remains inverse to CSTIII and IV, but the magnitude is substantially attenuated in adjusted models and both Wald ps> 0.10. Removal of educ_dich from adjusted models does not indicate confounding via change in coefficient for other variables

////////////////////////////////////////

*poormhm NS in adjusted model, becomes attenuated and NS in the presence of: age, education, HSV, median paid

*not affected by nonpaying partner, # sex partners, STI status, hormonal contraceptive use, days since last sex, difficulty accessing water at home or work, difficulty accessing privacy, where meet sex partners

FINAL MODEL

mlogit cst134 poormhm calcage i.numsp30d2 medianpaid i.hormonal2 sti hsv if read>2500, r rr base(1)

/////////////////ALPHA DIVERSITY

1. Model: Factors associated with evenness

aic_model_selection regress evenness poormhm bv sti hsv i.hormonal2 medianpaid daysince q24 calcage q59_5 q88 educ_dich q95 q110_reco q61_a if read>2500

AIC Model

-1780.799 evenness poormhm

-1882.147 evenness poormhm bv

-1899.134 evenness poormhm bv sti

-1897.007 evenness poormhm bv sti hsv

-1903.531 evenness poormhm bv sti hsv i.hormonal2

-1908.262 evenness poormhm bv sti hsv i.hormonal2 medianpaid

-1908.404 evenness poormhm bv sti hsv i.hormonal2 medianpaid daysince

*-1911.392 evenness poormhm bv sti hsv i.hormonal2 medianpaid daysince q24

*-1911.325 evenness poormhm bv sti hsv i.hormonal2 medianpaid daysince q24 calcage

*-1910.708 evenness poormhm bv sti hsv i.hormonal2 medianpaid daysince q24 calcage q59_5

-1909.242 evenness poormhm bv sti hsv i.hormonal2 medianpaid daysince q24 calcage q59_5 q88

-1907.772 evenness poormhm bv sti hsv i.hormonal2 medianpaid daysince q24 calcage q59_5 q88 educ_dich

-1905.992 evenness poormhm bv sti hsv i.hormonal2 medianpaid daysince q24 calcage q59_5 q88 educ_dich q95

-1904.25 evenness poormhm bv sti hsv i.hormonal2 medianpaid daysince q24 calcage q59_5 q88 educ_dich q95 q110_reco

-1902.397 evenness poormhm bv sti hsv i.hormonal2 medianpaid daysince q24 calcage q59_5 q88 educ_dich q95 q110_reco q6 1_a

-1824.143 evenness poormhm sti i.hormonal2 q24 hsv calcage medianpaid q59_5 educ_dich q110_reco q9_reco q61_a

-1824.13 evenness poormhm sti i.hormonal2 q24 hsv calcage medianpaid q59_5 educ_dich q110_reco q9_reco q61_a q88

-1821.136 evenness poormhm sti i.hormonal2 q24 hsv calcage medianpaid q59_5 educ_dich q110_reco q9_reco q61_a q88 daysince

-1819.869 evenness poormhm sti i.hormonal2 q24 hsv calcage medianpaid q59_5 educ_dich q110_reco q9_reco q61_a q88 daysince q95

*Comparing top models, the one including calcage is more informative. Calcage is not significant wald p-value but appears to have confounding affect on several variables having change in coefficient >10%. Examining addition of q95_5 (sex den/brothel) Wald p-value is not significant, and does not have confounding effect on other covariates.

FINAL MODEL

regress evenness poormhm bv sti hsv i.hormonal2 medianpaid daysince q24 calcage if read>2500, r

1. Factors associated with richness

aic_model_selection regress richness poormhm bv hsv sti daysince outside q24 educ_dich i.hormonal2 q59_5 q59_4 medianpaid q59_7 nonpaying calcage q61_a if read>2500

AIC Model

1557.626 richness poormhm

1506.577 richness poormhm bv

1480.857 richness poormhm bv hsv

1464.848 richness poormhm bv hsv sti

1447.976 richness poormhm bv hsv sti daysince

1441.075 richness poormhm bv hsv sti daysince outside

1434.692 richness poormhm bv hsv sti daysince outside q24

1429.665 richness poormhm bv hsv sti daysince outside q24 educ_dich

1426.187 richness poormhm bv hsv sti daysince outside q24 educ_dich i.hormonal2

1420.851 richness poormhm bv hsv sti daysince outside q24 educ_dich i.hormonal2 q59_5

1418.917 richness poormhm bv hsv sti daysince outside q24 educ_dich i.hormonal2 q59_5 q59_4

1416.498 richness poormhm bv hsv sti daysince outside q24 educ_dich i.hormonal2 q59_5 q59_4 medianpaid

1415.206 richness poormhm bv hsv sti daysince outside q24 educ_dich i.hormonal2 q59_5 q59_4 medianpaid q59_7

* 1414.939 richness poormhm bv hsv sti daysince outside q24 educ_dich i.hormonal2 q59_5 q59_4 medianpaid q59_7 nonpaying

* 1414.65 richness poormhm bv hsv sti daysince outside q24 educ_dich i.hormonal2 q59_5 q59_4 medianpaid q59_7 nonpaying calcage

1415.764 richness poormhm bv hsv sti daysince outside q24 educ_dich i.hormonal2 q59_5 q59_4 medianpaid q59_7 nonpaying calcage q61_a

Comparing 2 models with comparable AIC: There are 2 variables with wald p >0.10 - nonpaying and calcage. Removal of nonpaying (wald p=0.192) does not change coefficients of any other variables. Removal of calcage demonstrates confounding with several variables as reflected in coefficient change >10% , so maintain in model.

Final model:

regress richness poormhm bv hsv sti daysince outside q24 educ_dich i.hormonal2 q59_5 q59_4 medianpaid q59_7 calcage if read>2500, r

poormhm is attenuated and non-significant with HSV, and partially attenated by q59_5, median paid, educ_dich though remains marginal significant

poormhm association with richness is not affected by outside work or daysince lmp

# Supplemental Results: Screening and Enrollment Flow, POWWeR Health Study


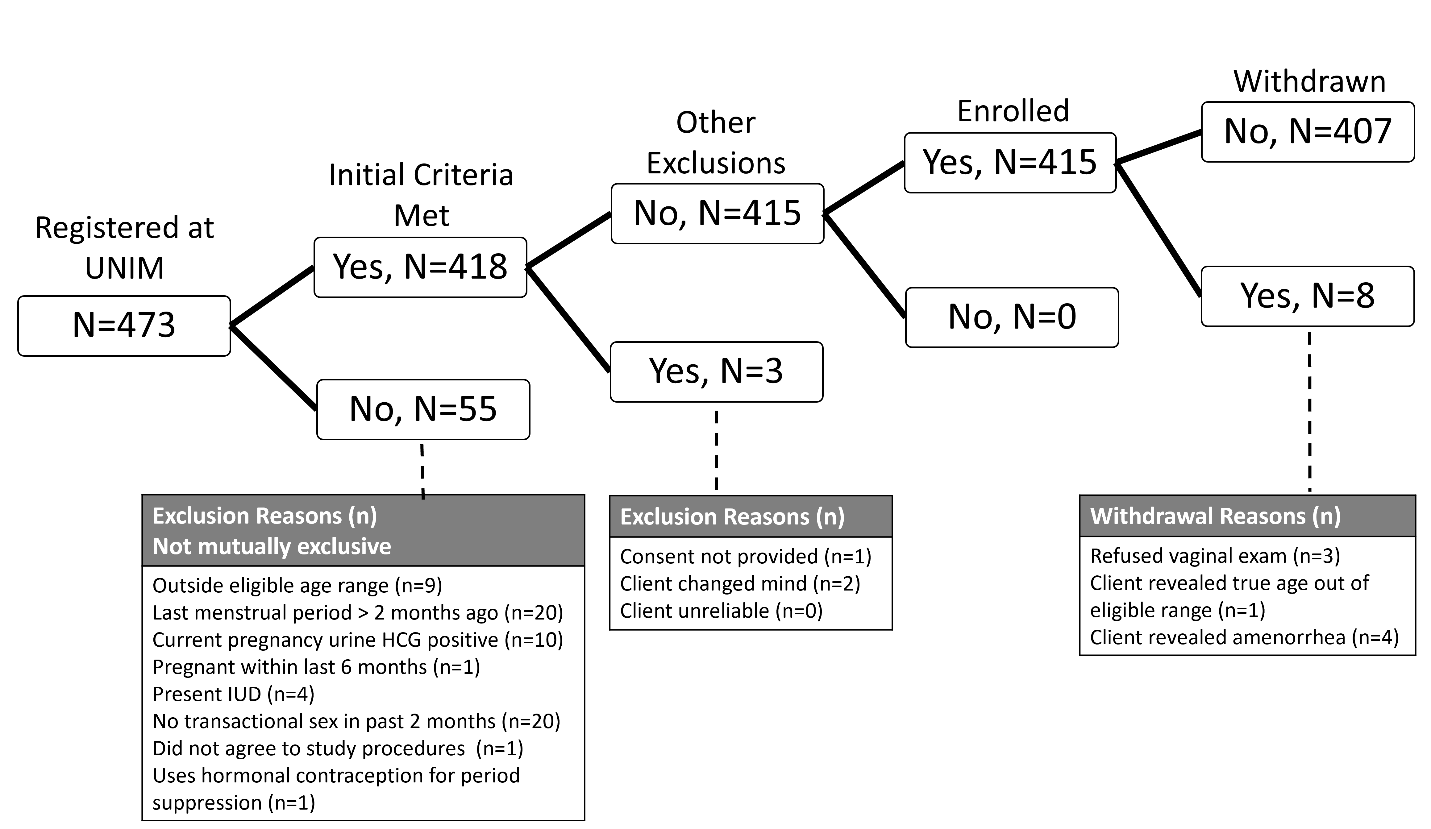


Women could be ineligible for more than one reason. Overall, 55 women did not meet initial eligibility criteria: having last menstruated more than 2 months ago (n=20), not having had sex in exchange for basic needs in the past 2 months (n=20), being outside the age range (n=9), not agreeing to study procedures (n=1), being recently post-partum (n=1), having an IUD (n=4), being currently pregnant via urine HCG test (n=10), using hormonal contraception for period suppression (n=1).

Subsequently, of 418 women meeting initial enrolment criteria, one declined consent and two changed their mind about study participation prior to initiating the consent process. Of 415 women consented, there were 8 subsequent screen failures: 4 revealed amenorrhea during the course of the baseline visit, 1 revealed true age to be outside of the eligible range, and 3 refused vaginal examination.

**Supplemental Table 1. Factors associated with using sub-optimal materials to manage menses**

|  | **Adequate Menstrual Materials***  **N=231**  **n (%)** | **Sub-Optimal Menstrual Materials (Cotton, Tissues, Cloth)**  **N=167**  **n (%)** | **P-value** | **Prevalence Ratio (95% CI)** | **Adjusted Prevalence Ratio (95% CI)**  **N=398** |
| --- | --- | --- | --- | --- | --- |
| Median Age (IQR) | 25 (22 – 30) | 29 (25 – 32) | <0.001 | 1.06 (1.04 – 1.09) | 1.03 (1.01 – 1.05) |
| Educational attainment   Below secondary school  Secondary school or higher | 113 (48.3)  118 (72.0) | 121 (51.7)  46 (28.0) | <0.001 | ref  0.54 (0.41 – 0.71) | ref  0.70 (0.53 – 0.91) |
| Income in past one month  <5,000 KSH  5,000 - <10,000 KSH  >10,000 KSH | 17 (54.8)  94 (56.3)  120 (60.0) | 14 (45.2)  73 (43.7)  80 (40.0) | 0.720 |  |  |
| Missed a meal past 7 days  No  Yes | 217 (61.6)  14 (30.4) | 135 (38.4)  32 (62.6) | <0.001 | ref  1.81 (1.44 – 2.28) | ref  1.31 (1.04 – 1.64) |
| Has income outside of sex work  No  Yes | 123 (52.8)  108 (65.5) | 110 (47.2)  57 (34.5) | 0.012 | ref  0.69 (0.55 – 0.88) | ref  0.78 (0.61 – 0.99) |
| Non-paying boyfriend or husband  No  Yes | 64 (53.3)  167 (60.1) | 56 (46.7)  111 (39.9) | 0.211 |  |  |
| Paid 700 KSH or more at last sex act  Paid <700 KSH  Paid > 700 KSH | 98 (50.3)  133 (65.5) | 97 (49.7)  70 (34.5) | 0.002 | ref  0.69 (0.55 – 0.88) |  |
| Median (IQR) number of sex partners, past 30 days  10 partners or less  11-20 partners  21-30 partners  31-50 partners  More than 50 partners | 20 (10 – 40) | 40 (20 – 60) | <0.001 | ref  1.57 (0.96 – 2.57)  2.15 (1.29 – 3.61)  2.38 (1.59 – 3.79)  2.80 (1.77 – 4.44) | ref  1.56 (0.98 – 2.48)  1.87 (1.15 – 3.08)  2.08 (1.32 – 3.27)  2.06 (1.31 – 3.26) |
| Difficulty accessing privacy at home  No  Yes | 210 (60.3)  21 (42.0) | 138 (39.6)  29 (58.0) | 0.014 | ref  1.46 (1.12 – 1.91) |  |
| Difficulty accessing privacy at sex work  No  Yes | 207 (63.9)  24 (32.4) | 117 (36.1)  50 (67.6) | <0.001 | ref  1.87 (1.51 – 2.32) | ref  1.38 (1.11 – 1.72) |
| Where meets clients  In street  No  Yes  In a home  No  Yes  At a truck stop  No  Yes  In a sex den or brothel  No  Yes  In a bar/club/restaurant  No  Yes  Lodge/hotel/guest house  No  Yes  On the internet  No  Yes | 88 (61.5)  143 (56.1)  216 (58.2)  15 (55.6)  218 (59.4)  13 (41.9)  186 (64.6)  45 (40.9)  69 (60.0)  162 (57.2)  121 (65.4)  110 (51.6)  168 (57.5)  63 (59.4) | 55 (38.5)  112 (43.9)  155 (41.8)  12 (44.4)  149 (40.6)  18 (58.1)  102 (35.4)  65 (59.1)  46 (40.0)  121 (42.8)  64 (34.6)  103 (48.4)  124 (42.5)  43 (40.6) | 0.290  0.786  0.058  <0.001  0.614  0.006  0.734 | ref  1.69 (1.34 – 2.08)  ref  1.40 (1.10 – 1.78) | ref  1.24 (0.98 – 1.56)  ref  1.29 (1.03 – 1.62) |

*Menstrual management material was not assessed for n=9 participants who reported not having a period in the past 6 weeks.

**Supplemental Table 3. Analysis of similarities for differences between groups based on Bray Curtis resemblance**

| **Groups: Sub-Optimal Menstrual Materials (MM) and Infection Status in Comparison to Negative for All Infections with Adequate MM** | **R**  **Statistic** | **Significance Level** |
| --- | --- | --- |
| Sub-Optimal MM vs. Adequate MM | 0.057 | 0.135 |
| BV vs. Negative | 0.587 | 0.001 |
| HSV-2 vs. Negative | 0.041 | 0.024 |
| BV+HSV-2 vs. Negative | 0.69 | 0.001 |
| HIV+HSV-2 vs. Negative | 0.223 | 0.001 |
| BV+HIV+HSV-2 vs. Negative | 0.737 | 0.001 |
| BV+STI vs. Negative | 0.636 | 0.001 |
| BV+STI+HSV-2 vs. Negative | 0.638 | 0.001 |
| BV, STI, HIV, HSV-2 vs. Negative | 0.329 | 0.001 |

Sequence counts were ln(x+1) transformed prior to Bray Curtis estimation. R statistic based on 999 permutations with Spearman rank correlation.

**Supplemental Table 4. Results of similarity of percentages comparison: top taxa contributing to dissimilarity for sub-optimal menstrual materials (MM) and infection outcomes**

| Average dissimilarity = 50.59 | Adequate MM  No Infection  N=55 | Sub-Optimal MM  No Infection  N=25 | Adequate MM  No Infection  N=55 | Sub-Optimal MM  No Infection  N=25 | Average Dissimilarity | Dissimilarity  (%) | Cumulative  percent |
| --- | --- | --- | --- | --- | --- | --- | --- |
| Species | Mean RA (%) | Mean RA (%) | Present (%) | Present (%) |  |  |  |
| *Lactobacillus crispatus* | 22.6 | 16.4 | 55 (100) | 25 (100) | 2.35 | 4.65 | 4.65 |
| *Lactobacillus iners* | 38.9 | 31.3 | 55 (100) | 25 (100) | 1.80 | 3.57 | 8.22 |
| *Lactobacillus vaginalis* | 0.27 | 0.24 | 31 (56.4) | 15 (60.0) | 1.59 | 3.13 | 11.35 |
| *Ureaplasma parvum* | 0.34 | 0.21 | 31 (56.4 | 12 (48.0) | 1.50 | 2.97 | 14.32 |
| *Gardnerella vaginalis* | 9.40 | 16.8 | 55 (100) | 25 (100) | 1.43 | 2.84 | 17.16 |
| *Lactobacillus* (genus) | 0.58 | 1.04 | 51 (92.7) | 24 (96.0) | 1.43 | 2.83 | 19.99 |
| *Burkholderia_caballeronia_*  *paraburkholderia* | 4.97 | 3.82 | 54 (98.2) | 25 (100) | 1.41 | 2.79 | 22.78 |
| *Lactobacillus jensenii* | 1.55 | 1.14 | 27 (49.1) | 7 (28.0) | 1.34 | 2.64 | 25.41 |
| *Gardnerella* (genus) | 3.97 | 3.90 | 54 (98.2) | 23 (92.0) | 1.27 | 2.51 | 27.92 |
| *Veillonella montpellierensis* | 2.12 | 2.01 | 44 (80.0) | 20 (80.0) | 1.21 | 2.39 | 30.31 |
| *Atopobium vaginae* | 1.79 | 2.11 | 43 (78,2) | 17 (68.0) | 1.19 | 2.35 | 32.66 |
| *Streptococcus* (genus) | 2.06 | 1.85 | 48 (87.3) | 20 (80.0) | 1.15 | 2.28 | 34.94 |
| *Prevotella timonensis* | 1.74 | 2.37 | 41 (74.6) | 19 (76.0) | 1.14 | 2.25 | 37.19 |
| Veillonellaceae (family) | 1.47 | 2.63 | 43 (78,2) | 19 (76.0) | 1.14 | 2.25 | 39.44 |
| *Prevotella amnii* | 2.14 | 2.83 | 46 (83.6) | 24 (96.0) | 1.13 | 2.24 | 41.68 |
| *Aerococcus christensenii* | 1.28 | 1.8 | 31 (56.4) | 16 (64.0) | 1.13 | 2.22 | 43.9 |
| *Sneathia amnii* | 2.04 | 2.31 | 52 (94.6) | 20 (80.0) | 1.03 | 2.04 | 45.95 |
| *Lactobacillus gasseri* | 0.96 | 1.24 | 37 (67.3) | 11 (40.0) | 1 | 1.98 | 47.92 |
| Clostridiales (family) | 1.52 | 1.87 | 41 (74.6) | 19 (76.0) | 0.99 | 1.96 | 49.89 |

**Supplemental Figure 1. Proportional Venn Diagram Showing the Overlap of Using Sub-Optimal Menstrual Materials (Cotton Balls, Tissues, Cloth), Difficulty Accessing Water at Home and/or at Sex Work, and Difficulty Accessing Privacy at Home and/or at Sex Work**


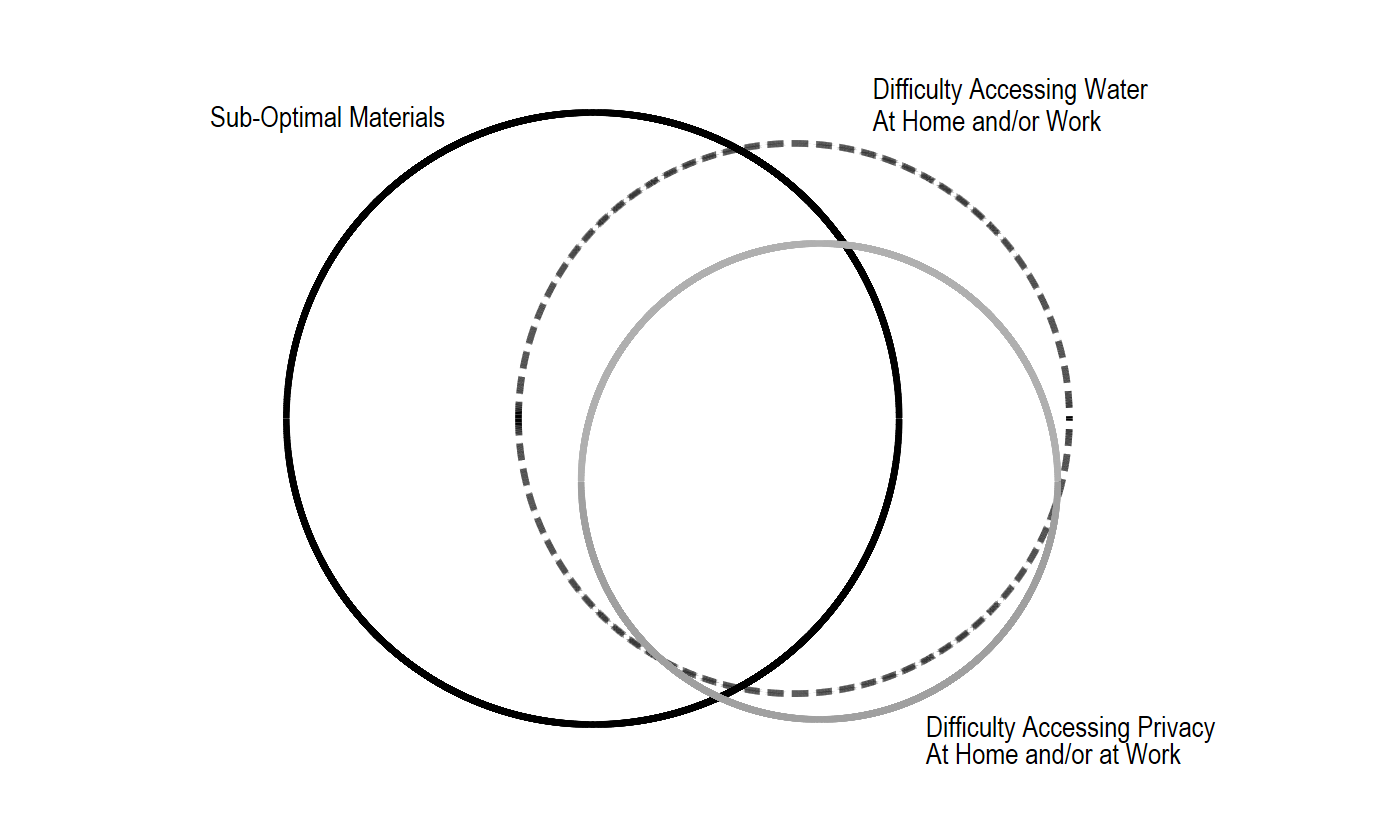


**Supplemental Figure 2. Distribution of alpha diversity metrics over infection and outcomes.**


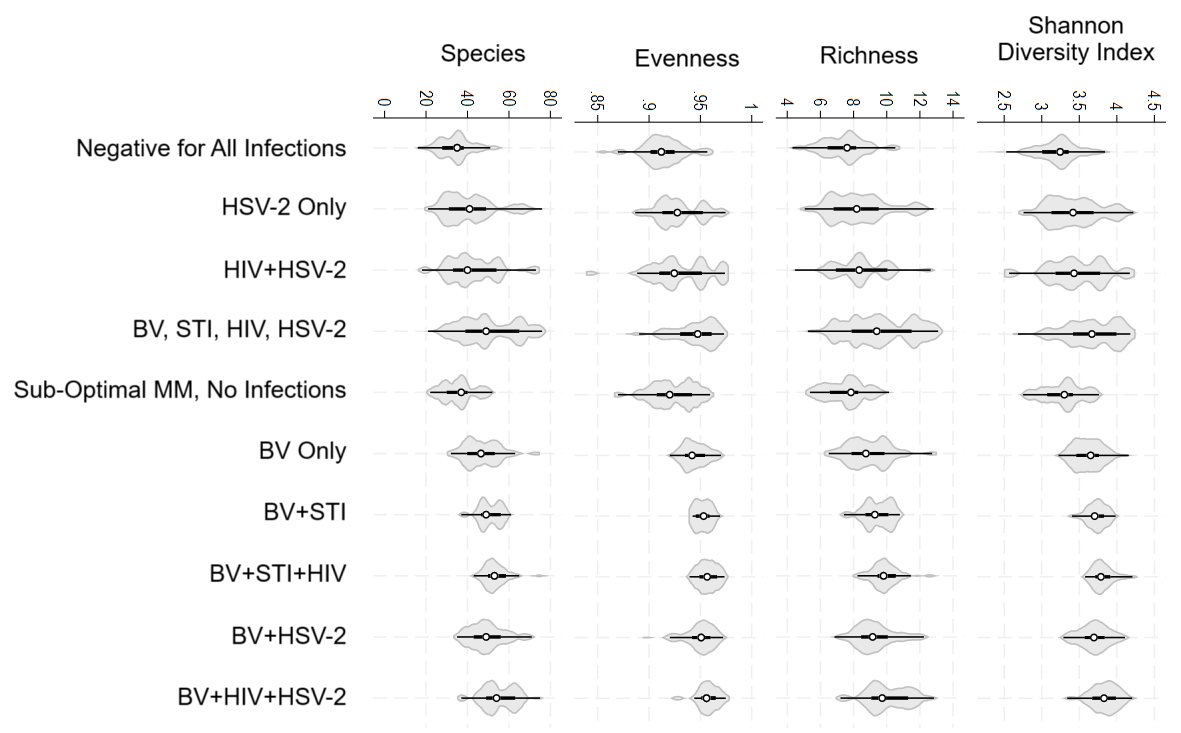


**Legend:** The distribution of alpha diversity metrics (Species index, Evenness, Richness, and Shannon diversity index) are shown at the top while the status of subjects is shown along the vertical. White, open circles represent medians with black bars representing interquartile ranges, and grey shading reflecting distribution densities.
